# Psychological resilience and competence: key promoters of successful aging and flourishing in late life

**DOI:** 10.1007/s11357-023-00856-9

**Published:** 2023-07-07

**Authors:** Virág Zábó, Anna Csiszar, Zoltan Ungvari, György Purebl

**Affiliations:** 1 Doctoral School of Psychology, Eötvös Loránd University, Budapest, Hungary; 2 Institute of Psychology, Faculty of Education and Psychology, Eötvös Loránd University, Budapest, Hungary; 3 Institute of Behavioural Sciences, Faculty of Medicine, Semmelweis University, Budapest, Hungary; 4 Vascular Cognitive Impairment, Neurodegeneration and Healthy Brain Aging Program, Department of Neurosurgery, University of Oklahoma Health Sciences Center, Oklahoma City, OK USA; 5 Oklahoma Center for Geroscience and Healthy Brain Aging, University of Oklahoma Health Sciences Center, Oklahoma City, OK USA; 6 International Training Program in Geroscience, Doctoral School of Basic and Translational Medicine/Department of Public Health, Semmelweis University, Budapest, Hungary; 7 Department of Health Promotion Sciences, College of Public Health, University of Oklahoma Health Sciences Center, Oklahoma City, OK USA

**Keywords:** Successful aging, Resilience, Competencies, Flourishing, Maintainable Positive Mental Health

## Abstract

Many individuals, both in the public and within the field of psychology, often perceive aging as a burden that negatively impacts intellectual and mental health. Our present study aims to challenge this notion by identifying the crucial components of positive mental health in later life. These components not only promote positive mental health but also actively contribute to it, even under difficult circumstances. To accomplish this, we first offer a concise review of well-being and mental health models that highlight the psychological aspects of flourishing in late life. We then introduce a psychological competence-based model for positive mental health, which aligns with the concept of positive aging. Subsequently, we present a measurement tool suitable for practical applications. Finally, we provide a comprehensive overview of positive aging, drawing on methodological guidelines and existing research findings concerning sustainable positive mental health in later life. We examine the evidence indicating that psychological resilience (the capacity to adapt and recover from adversity or stress) and competence (skills and abilities to effectively cope with challenges across various life domains) significantly contribute to slowing down biological aging processes. Furthermore, we discuss insights into the relationship between psychological factors and aging derived from research on Blue Zones (regions characterized by a higher proportion of individuals experiencing longer, healthier lives).

## Introduction

The population of the western world is aging at an unprecedented rate. This demographic shift is driven by several factors, including improvements in healthcare, resulting in increased life expectancy. As a result, the number of Americans ages 65 and older will more than double over the next 40 years, reaching 80 million in 2040 [1]. In the European Union, the proportion of people over 65 years old is expected to rise sharply until 2058, accounting for 30.3% of the population [2]. The Asia–Pacific region is projected to experience the fastest increase in older citizens, with one in four individuals being over 60 years old by 2050, resulting in 1.3 billion senior citizens [3]. According to the World Health Organization, the number of individuals aged 60 or older globally is expected to double by 2050, reaching an astounding two billion [4].

With the growing population of older adults, the concept of “successful aging” has become increasingly important. From a geroscience perspective, successful and healthy aging involves the optimization of biological aging processes over the course of a person’s lifetime. This can lead to a reduction in the rate of age-related functional decline, resulting in a delayed onset of age-related diseases and a longer maintenance of functional ability. This, in turn, enables well-being in older age, as defined by the World Health Organization’s concept of “healthy aging” [5]. To guide this study, we adopt the concept first introduced by Rowe and Kahn, which defines successful aging as the attainment of high levels of physical, psychological, and social functioning in old age, coupled with the absence of significant age-related diseases [6–8]. This concept emphasizes the importance of enabling older adults to remain active and productive members of society, rather than simply viewing aging as a period of decline and dependency.

There are multiple reasons why promoting successful aging is crucial. Firstly, it can improve the health and well-being of older individuals, resulting in a better quality of life and reduced pressure on healthcare systems. Secondly, it can be advantageous for society as a whole by enabling older adults to continue contributing to their communities, be it through paid or voluntary work, or supporting family members. Lastly, promoting successful aging can combat ageism and negative stereotypes about aging, which often restrict the opportunities available to older adults. Overall, given the demographic changes taking place, promoting successful aging is an important social and economical necessity for the communities. By supporting the health, well-being, and productivity of older adults, we can create a more inclusive and equitable society for all age.

To comprehensively understand successful aging, a multidisciplinary approach is necessary, as illustrated by the wide range of scientific fields covered in this journal. Here we focus on the physiological aspects of successful aging. Over the years, various cross-sectional and longitudinal psychological studies [9–14] have been conducted to investigate the nature of successful aging. These studies examine cognitive [15] and health [10, 11, 16] aspects, among others [11]. This approach has become increasingly important due to the growing elderly population resulting from increased life expectancy. As a result, promoting the well-being of older adults has become a key strategic goal for social and health policymakers worldwide, as highlighted by the World Health Organization [17].

Given the significant interest in investigating well-being in later life, understanding the factors that contribute to successful aging and complete physical and mental well-being of older adults is critical. Although “aging well” is a target state, it is surrounded by many concepts in addition to successful aging [18, 19], such as active aging [20, 21], productive aging [22], vital aging [23], optimal aging [18], healthy aging [24], harmonic aging [18], or simply good aging [9]. Despite the popularity of the successful aging construct in gerontology, there is no consensus regarding its definition.

While from a geroscience perspective it is an accurate and useful term, in the field of psychology the scientific construct of successful aging has often been criticized for its emphasis on values. The term “successful” is often associated with connotations such as fame or respect, which can create the perception that those who do not meet the criteria for successful aging are failures. As a result, the concept of positive aging has emerged as an alternative approach that encompasses the essence of successful aging while avoiding these negative associations. Positive aging focuses on the content of aging, emphasizing everything that constitutes a positive and fulfilling experience in later life. The concept of positive aging does not imply that certain achievements or values must be met in order to be considered successful. Instead, it highlights the importance of factors such as maintaining physical and mental health, maintaining social connections, and pursuing meaningful activities in later life. Overall, emphasizing the positive aspects of aging can help to combat ageism and negative stereotypes about aging, promoting a more positive and inclusive view of later life.

## Positive psychology and aging

The concept of successful aging aligns closely with the mission statement of positive psychology, which is to study flourishing, optimal human functioning, and well-being [25]. Positive psychology places a strong emphasis on identifying indicators of mental health that contribute to long-term well-being, creativity, and flourishing, and developing theoretical frameworks to understand positive mental health. This approach differs from the traditional psychopathology-focused approach to mental health, as it prioritizes a person’s strengths and resources [25]. While positive psychology has been effective in examining the flourishing of healthy individuals, there has been a lack of focus on how it can apply to the life experiences of vulnerable individuals [26], such as older adults. The implication of this positive-focus model has significant potential in the field of gerontology, particularly in promoting late-life well-being and productivity. By seeking to identify, find, and enhance positive psychological changes and resources, instead of merely minimizing the negative effects of psychological frailty in aging [27, 28], the positive psychology model can offer a more effective approach to aging.

While there are challenges in applying positive psychology to aging, such as theoretical and methodological difficulties, ongoing research in this field continues to expand our knowledge of well-being and mental health. Despite the multiple physical and social losses associated with aging, subjective well-being (SWB) is stable or even increasing in later life [29]. This highlights the importance of identifying the positive components of mental health in late life rather than conceptualizing mental health based solely on the absence or presence of psychopathology. However, while positive psychology has provided valuable insights into well-being and mental health, the field must still address theoretical and methodological challenges in developing new theories and measurement tools. These challenges include psychometric difficulties and the need for more empirical research to support the validity of positive psychology concepts in the context of aging [30–32].

Overall, positive psychology offers a valuable framework for promoting well-being in older adults by focusing on their strengths and resources rather than merely on their deficits and limitations. By emphasizing the positive aspects of aging, this approach can help individuals achieve optimal functioning and a fulfilling life in later years.

## Previous models of well-being and mental health in late life

Previous models of well-being and mental health in late life have explored various conceptualizations, including (1) multidimensional well-being [33–36], (2) mirror opposite to the symptoms of mental disorders [37–39], (3) flourishing [34], (4) “hedo-eudemonic” well-being [33, 38, 40–43], (5) classical models of mental health [44–51], (6) balanced models of mental health [52–55], and (7) the sum of the components of well-being [33, 39, 40, 42, 43, 56, 57]. While these models have focused on the components of well-being, they have not fully captured all aspects of mental health as defined by the World Health Organization (WHO) and classical theories of mental health. The WHO defines mental health as a dynamic state of internal equilibrium that enables individuals to use their abilities in harmony with universal values of society. Components of mental health include basic cognitive and social skills; the ability to recognize, express, and modulate emotions; empathy; flexibility; coping skills; social role functioning; and a harmonious relationship between body and mind, which contribute, to varying degrees, to the state of internal equilibrium [58]. Therefore, to fully capture mental health in late life, measurement tools should go beyond the operationalizations that define the concept with observable characteristics of well-being or as the mirror opposite of mental disorders. Incorporating the concept of successful aging can provide a more comprehensive approach to understanding and promoting mental health in late life.

## Conceptual and methodological difficulties of well-being and mental health measurements

Measuring well-being and mental health presents significant conceptual and methodological challenges for positive psychology [59]. Inconsistent factor structures, varying internal consistency ranges, and differences in predictive ability across cultures have been found in different positive psychology measures [32]. Recent criticisms of positive psychology [30, 32] have highlighted the fallibility of measuring instruments, directly affecting the credibility of the discipline and its underlying theories. Furthermore, the heterogeneity and multitude of psychological concepts and theories that fall under the umbrella of positive psychology make it difficult to compare research results based on constructs with different contents. Mixing predictor and indicator variables in cross-sectional and longitudinal studies on successful aging further confuses the methodology. Thus, there is a need to reconsider positive psychology models and to reconceptualize factors responsible for maintaining positive mental health in advanced age.

## The concept of psychological immune competence and its role in the maintenance of positive mental health in late life

Just as our body has an immune system to defend against harmful biological agents, our mind also requires psychological immune competence for stress resistance and resilience [51]. Unlike the effects of vaccines, these psychological skills and competencies are deep-wired and long-lasting, acquired over time. Beside than using the concept of successful aging, which has a value-laden connotation, we also propose maintainable (moreover, promotable) positive mental health as a practical, measurable indicator of positive aging. According to the Maintainable Positive Mental Health Theory originally developed by Oláh [60, 61], the level of well-being in later life depends on both the presence of psychological resources for positive mental health and the ability to utilize them effectively. This approach considers all theory-based but empirically identified components of well-being as features of mental health that reflect the presence and proper functioning of psychological capacities needed to maintain and promote positive psychological status and mental and physical health. This is in line with the World Health Organization’s definition of mental health as a dynamic state of internal equilibrium that enables individuals to use their abilities in harmony with universal values of society [58]. In addition to the components of well-being, resilience, accommodation to changes, and the development of efficient coping capacities such as savoring and establishing positive states while handling negative states are also major contributors to positive mental health. Therefore, our suggested definition of mental health in later life includes a high level of global well-being; psychological, social, and spiritual well-functioning; resilience; efficient creative and executive functioning; coping; and savoring capacities. These pillars ensure that individuals can flourish amidst the changes and challenges along with the age-related psychological frailty, including declines, losses, and negative events. The quality of these protective factors is decisive in strengthening an individual’s coping capacity and guaranteeing flourishing despite potential negative events, challenges, health issues, or losses during the aging process.

## Mental Health Test — a comprehensive quantitative measure of psychological strengths and resources

The Mental Health Test [60] serves as the operationalized, comprehensive measurement of Maintainable Positive Mental Theory (MPMHT) [61]. The first pillar of MPMHT is Global Well-being, which integrates existing well-being theories and encompasses multi-component subjective well-being in emotional, psychological, social, and spiritual areas of life [43, 62, 63]. Table 1 outlines the pillars of Global Well-being and their relationship with self-regulation, savoring capacity, resilience, and creative and executive efficiency.

**Table 1:** Pillars of Global Well-being according to Maintainable Positive Mental Health Theory (adapted from ref. [60])

Global well-being			
Emotional well-being	Positive functioning		Spiritual well-being
	Psychological well-being	Social well-being	
Positive affect Happiness Life satisfaction	Self-acceptance Personal growth Environmental mastery Autonomy Positive relations with others	Social acceptance Social actualization Social contribution Social coherence Social integration	Joy of transcendence experience Joy of universality experience Vertical and horizontal responsibility

Note that the pillars of global well-being refer to the set of features of mental health that reflect the presence and proper functioning of the psychological capacities needed to maintain and promote positive psychological status and mental health, according to the Maintainable Positive Mental Health Theory

Savoring is the second pillar, referring to the ability to mentally relive joyful memories and experiences, generating mental well-being and extending it to future events [64]. Savoring is a necessary ability for MPMHT, as it contributes to achieving and maintaining positive mental health [65].

The third pillar is Creative and Executive Efficiency, which enables individuals to cope with difficulties and challenges by mobilizing their competencies in individual and social problem-solving [43, 51].

The fourth pillar is Self-regulation, the ability to regulate and control temperament, emotions, and negative states while persisting in achieving a goal. This ability plays a critical role in mental health and represents one of the most adaptive variables of human behavior [66–68].

Finally, Resilience is the fifth pillar, referring to an individual’s psychological capacity to mobilize their resources and maintain positive mental health when facing unexpected, stressful situations. The higher the level of resilience, the more quickly the individual can recover from such situations [69–71].

According to MPMHT, these five pillars are responsible for an individual’s mental health. The competencies associated to the five pillars can be trained, improved, and strengthened. Thus, the pillars provide an easy-to-follow concept for the aging population. Firstly, it provides a structural model for assessing the individual capacities and resources (personal sources of resilience, the person’s own creativity, and his/her executive competencies as well as the sources of peer support and social connectedness). Secondly, based on this assessment the aging person can work up an equilibrium with their own physical and mental status as well as with the outside world, by promoting his/her development, creating a steady state for personal and social functioning (self-regulation), and an equilibrium of positive and negative emotions (coping, savoring). The mindful application of the five-pillar model and therefore the existence and efficient functioning of these elements may improve mental and physical well-being and social functioning, may increase the level of spiritual connectedness, and, through the preservation/promotion) of mental and physical help and global functioning, eventually might contribute to delay aging.

## Psychological resilience, competence, and slowing biological aging processes: insights from Blue Zone studies

Research on the Blue Zones, regions where a higher proportion of individuals live longer and healthier lives, offers valuable insights into the connection between psychological factors and aging [72]. Blue Zones include five areas: Okinawa (Japan), Sardinia (Italy), Nicoya (Costa Rica), Icaria (Greece), and the Seventh-day Adventist community in Loma Linda (CA, USA) [73–77]. Studies conducted in these regions have identified several commonalities that contribute to longevity, including lifestyle, diet, and social factors [72–74, 78–82]. These findings have been confirmed by subsequent studies in different populations [83, 84]. Individuals living in the Blue Zones have been observed to exhibit a younger biological age compared to their chronological age [85]. This region-specific slowing down of aging processes is attributed to the unique lifestyle, dietary, and social factors prevalent in these regions.

Blue Zone populations are a valuable resource for the study of positive aspects of aging. Importantly, psychological resilience and competence likely play a significant role in slowing down biological aging processes, contributing to overall well-being and having a positive impact on physical health and longevity [77, 86, 87]. One key insight from Blue Zone research is the importance of a strong sense of purpose, known as “ikigai” [88, 89] in Okinawa and “plan de vida” in Nicoya [73]. A well-defined purpose in life contributes to psychological resilience and competence, as it fosters motivation, determination, and a positive outlook. This sense of purpose is thought to reduce stress and anxiety and increase the sense of connectedness, which can help protect against the harmful effects of chronic stress on biological aging processes [83, 84].

Drawing inspiration from lessons learned through Blue Zone research, individuals may benefit from a structured approach to discovering meaning in their lives, such as through targeted interventions. In this context, the concept of “life crafting” was introduced, a process grounded in positive psychology and the salutogenesis framework [90–93]. Life crafting typically begins with an intervention that incorporates a blend of self-reflection on values, passions, and objectives, envisioning one’s best possible self, developing goal attainment strategies, and employing other positive psychology techniques. Crucial components of such an intervention include identifying values and passions, examining current and desired abilities and habits, contemplating present and future social connections, outlining specific goal achievement, and committing to the established goals [92]. Previous research has demonstrated that personal goal setting and goal attainment strategies can provide individuals with direction and a sense of purpose [92]. By drawing from research findings in positive psychology, such as salutogenesis, sense of coherence, implementation intentions, value congruence, broaden-and-build, and goal-setting literature, a comprehensive, evidence-based life-crafting intervention program can be developed [92]. Informed by insights from the Blue Zones, this intervention can assist individuals in identifying their life purpose while concurrently ensuring that they create tangible plans to pursue it. The underlying premise is that life crafting empowers individuals to take charge of their lives, ultimately optimizing performance and happiness.

Another aspect highlighted in Blue Zone studies is the value of social connections and support networks [72]. Maintaining close relationships with family, friends, and community members can promote psychological resilience and competence by both accepting and providing emotional support, encouragement, and a sense of belonging. Social connections also facilitate the exchange of knowledge, resources, and coping strategies, which can further enhance an individual’s ability to deal with challenges and stressors.

Furthermore, the Blue Zones emphasize the role of regular physical activity and a predominantly plant-based diet [78, 81, 82, 94–96], which have been linked to improved mental health, increased cognitive functioning, and reduced risk of age-related diseases. By maintaining a healthy lifestyle, individuals can foster their psychological resilience and competence while simultaneously mitigating the negative impact of biological aging.

Taken together, psychological resilience and competence are essential factors in slowing down biological aging processes, as demonstrated by the Blue Zone studies. Cultivating a sense of purpose, nurturing social connections, and adopting a healthy lifestyle can help enhance these psychological factors and contribute to a longer, healthier life.

## Maintainable Positive Mental Health in late life

In this section we provide evidence and illustrate empirically proven strategies for the development of psychological immunocompetencies among the elderly. Emotional well-being can be improved in old age through various methods. Formal volunteering [97] is a low-cost and organic method that not only contributes to the more efficient functioning of a community but also gives older adults meaning and purpose while strengthening their commitment and social belonging. Another effective technique is to encourage older adults to be mindful of positive experiences regularly. For example, a study indicated that setting aside 5 min in the morning and 5 min in the evening each day for 1 week significantly increased the subjective well-being of participants [98]. Purposeful activity interventions, particularly those that involve taking on a functional role, can also improve well-being and quality-of-life outcomes in older adults aged 80 years and older [99].

Digital technology can play a crucial role in maintaining positive mental health in later stages of life. Recent studies have shown that the use of mobile fitness technology is associated with improved physical and psychological well-being, as measured by PERMA, among individuals aged 60 years or older [100]. Additionally, digital media can foster a sense of belonging and enhance communication among the elderly, leading to positive social outcomes [101].

Spiritual well-being is a crucial factor in maintaining positive mental health for older adults. Social innovation has been shown to contribute to enhancing the meaning and purpose of daily life for nursing home residents [102]. In a recent study, the process of spiritual care was found to involve identifying the spiritual needs and resources of older adults in healthcare, understanding their specific requirements, developing a personalized spiritual care treatment plan, and engaging relevant healthcare and spiritual care professionals to facilitate personal connections with meaning-making agents [103].

Other studies have demonstrated that mindfulness practices can have a positive impact on the happiness and resilience of adults aged 60 and over [98]. Practicing mindfulness by being aware of positive experiences and recognizing associated positive emotions twice a day for 5 min was found to improve well-being post-intervention, as well as 1 and 3 months later. Additionally, a 30-min mindfulness intervention, where participants were asked to savor positive emotions associated with connections with others, was found to enhance psychological agency in adults aged 60 to 90 years [104].

Research has shown that emotional intelligence-based interventions, including psychoeducation, can significantly improve the skills of older adults and increase their scores on resilience and life satisfaction [105]. Moreover, alternative interventions such as mindfulness and physical activity interventions have also been found to strengthen resilience in older adults [106].

Regular practice of moderately intense physical activity is a low-cost strategy that can help improve and maintain self-regulation. In one study, the mental well-being of 58 individuals aged 67 to 85 who participated in two moderately intense physical training programs was longitudinally evaluated [107]. The participants reported significantly more adaptive emotion regulation strategies after the intervention.

Additionally, self-efficacy can be improved among older adults through problem-solving therapy. An 8-week-long intervention was found to increase self-efficacy in elderly nursing home residents both at the end of the intervention and 3 months post-intervention [108].

These findings underscore the importance of early intervention, preventive community-based approaches, and the promotion of mental health for older adults. Public mental health conceptual frameworks, such as socioecological models, highlight the impact of individual, community, family/relational, and structural determinants [109, 110]. Jopling’s model can be a promising framework for planning future interventions, identifying which levels and agents can contribute to the mental health of the elderly [111].

To help older individuals maintain their positive mental health status, it is crucial to establish sustainable prevention and protection strategies. One such strategy is to leverage modern technological developments customized for older adults, which can be a key element in innovation practices. With increasing interest and proficiency in digital media platforms and applications, older adults can stay healthy, independent, and socially connected. Digital media can broaden their social networks, enable communication with peers and younger generations, organize social and group events, promote diversity, and facilitate exchange of social support, ultimately bringing about a positive change in their social network, connectedness, and social inclusion. Therefore, short online cost-effective interventions should be more boldly applied among them.

Spirituality is an internal psychological resource that can be accessed anywhere, anytime, by the elderly regardless of their life situation, circumstances, cognitive status, or physical complaints. Spirituality can be experienced in many forms, from religious practice to simple sense of coherence and connectedness, and can also be manifested in secular frameworks. It is important to tailor spiritual practices to individual needs and implement them in social constructs that are personalized for older adults.

Nursing homes need to become more homelike and patient- and family-centered to enhance the quality of life, satisfaction, and autonomy of their residents. Empirical evidence has shown that cultural changes in nursing homes, such as individualized care, meaningful relationships, opportunities for participation in life roles, and a sense of belonging, have had positive effects on residents. Therefore, it is important to continue and strengthen this cultural shift in nursing homes.

In recent years, positive psychological interventions have faced significant scrutiny. The Best-Practice Guidelines for Positive Psychological Intervention Research Design offers comprehensive guidelines that address crucial aspects of intervention methodology, including (1) intervention design, (2) participant recruitment and retention, (3) adoption, (4) fidelity and implementation concerns, and (5) efficacy or effectiveness evaluation [112]. Additionally, the Intervention Mapping Approach and Theoretical Domains Framework deliver detailed insights into the scientific development process of mental health interventions [113]. By doing so, they demonstrate how utilizing these methodologies can enhance the reporting standards for intervention development.

Positive psychology approaches can play a crucial role in alleviating the psychological burden experienced by the elderly during the COVID-19 pandemic [114] as well. By promoting positive emotions, enhancing resilience, and fostering a sense of purpose and meaning, these interventions can help older adults cope with the challenges of the pandemic and maintain their mental well-being. In addition, it is important to note that the older adults are particularly vulnerable to COVID-19 mortality and morbidity [115–119], which can exacerbate existing psychological distress [120–122]. Therefore, positive psychology interventions are not only important for maintaining mental health but can also have potential benefits for physical health outcomes in this population.

## Practical implications

The wider implementation MPMHT provides new perspectives for previous research on psychological immune competence and positive mental health [61] and a new conceptual and practical model for intervention programs aim to promote successful aging. Exploring and exploiting the existing (sometimes hidden) capacities of people previously focusing to age-related impairments not only help to shift their focus of attention. MPMHT not only helps to embrace the psychological frailty in aging as part of human experience but also provides purposeful practical strategies and techniques for promoting positive health and global functioning and, as a result, eventually may delay aging. The Mental Health Test (MHT) is the first test to have a five-dimensional complex structure covering a wide spectrum of mental health. The short completion time, the self-test design of the 18-item questionnaire, can provide the opportunity of measuring mental health competencies quickly and easily. It can be applied in epidemiological surveys, even in large-scale, representative survey programs, and also can serve as an everyday practical tool for the planning of the clients’ personalized health promotion interventions. Although various psychological measures are available, MHT’s unique structure comprehensively provides a panoramic view of the elderly’s mental health competencies, even among different kind of health care settings. Mental health institutions, nursing homes, social care institutions, and community-based services can adopt MHT into their daily practice. MHT serves for the comprehensive assessment of MPMHT but by the assessment of the resources of clients it also supports diagnostic work and the planning of personalized therapy. Beside therapy it can also help to improve the client’s level of global functioning when the goal is not primary symptom reduction but the restoration of daily functioning. Thus, MPMHT can propose a new paradigm for rehabilitation programs. The panoramic evaluation of resources and capacities with the structured assessment of the five pillars enables a more precise, personalized approach to promote the mental health of the elderly. The revealed personal resources can be used in counseling, therapy, and individual or group intervention programs. The prolonged maintenance of physical and mental functioning may have positive economic impact, since people living with mental disorders or other kind of disabilities may recover their functions more quickly, enabling them to maintain or improve their social productivity even in late life.

## Conclusion and perspectives

In conclusion, this paper has shed light on the persistent obstacle that impedes progress in understanding the vulnerability of the elderly, which remains entrenched in society and the field of psychology. However, flourishing older adults continue to experience personal growth as they evolve and change. Our review has highlighted that mental health components have well-defined features and competencies that are modifiable and therefore can be trained and strengthened. Creative and executive efficiency, self-regulation, and resilience show positive correlations with age, indicating their potential for improvement. Savoring also implies a mental ability that can be enhanced with cognitive techniques, ultimately leading to greater subjective well-being, life satisfaction, and happiness.

It is important to note that, despite the psychological frailty in aging (e.g., increasing losses and higher prevalence of age-related diseases), strengthening these competencies can promote autonomy and a sense of self-coherence. Future research should focus to the elaboration of the concept of positive, maintainable mental health in the elderly. The development and implementation of strategies of increasing resilience and decrease frailty can demonstrate that along with embracing of vulnerability, aging can also be a human experience of flourishing, connectedness, and coherence. Incorporating the growing number of interventions based on positive psychology (a short outline is presented in Table 2) into multi-domain approaches is essential for promoting successful aging, as they synergistically enhance psychological resilience and competence, contributing to overall well-being and longevity [123, 124].

**Table 2:** Categories of community interventions identified (adapted from ref. [123])

Intervention category	Description	Link to conceptual frameworks and determinants of mental health promotion
Connector interventions	Offer support to facilitate access and engagement with community resources, such as social activities or befriending services. Aim to reach individuals not presently involved with services or community activities, dedicate time to comprehend a person’s circumstances to provide a suitable response, and deliver practical and emotional support to access services	Focused on enhancing access to resources, engagement with support services, and addressing determinants of mental health promotion by understanding individual needs and circumstances
Gateway interventions	Infrastructure designed to aid older adults in connecting or staying connected with their community, ensuring that interventions and services are accessible and appropriate. Examples encompass the built environment, digital/technology resources, and community transport	Influenced by community-level drivers such as the economic built environment and community assets, which contribute to promoting mental health and well-being
Direct interventions (group-based or individual)	Support older adults to maintain and enhance social connections and relationships by directly supporting the formation of new connections, social activities, and offering psychosocial support to encourage changes in thinking and actions. Group-based interventions often center around creative or cultural themes, sometimes combined with group support or other social elements	Primarily influenced by community-level drivers, including social capital, with some individual-level drivers also playing a role in promoting mental health and well-being
System approaches	Focus on cultivating community environments that support older adults’ mental health by encouraging collaboration among key stakeholders in public mental health (e.g., local government, NHS, community, voluntary and faith sectors, local businesses). These efforts facilitate community-based actions tailored to local strengths, needs, and context. Initial outcomes often resemble outputs and processes, such as new groups, connections, networks, volunteering opportunities, awareness-raising, and tackling stigma. Interventions may incorporate community or asset-based approaches	Engages individual-level drivers (stigma and discrimination), community-level factors (social capital, assets), and potentially some structural drivers (e.g., commercial aspects, local norms, local economy) to promote mental health and well-being
